# MicroRNA Profiling Implies New Markers of Gemcitabine Chemoresistance in Mutant p53 Pancreatic Ductal Adenocarcinoma

**DOI:** 10.1371/journal.pone.0143755

**Published:** 2015-11-25

**Authors:** Sameer A. Dhayat, Wolf Arif Mardin, Jochen Seggewiß, Anda Jana Ströse, Christiane Matuszcak, Richard Hummel, Norbert Senninger, Sören Torge Mees, Jörg Haier

**Affiliations:** 1 Department of General and Visceral Surgery, University Hospital Muenster, Muenster, Germany; 2 Integrated Functional Genomics, Interdisciplinary Center for Clinical Research, Medical Faculty of the University of Muenster, Muenster, Germany; 3 Comprehensive Cancer Center Muenster, University Hospital Muenster, Muenster, Germany; Schulze Center for Novel Therapeutics, Mayo Clinic, UNITED STATES

## Abstract

**Background:**

No reliable predictors of susceptibility to gemcitabine chemotherapy exist in pancreatic ductal adenocarcinoma (PDAC). MicroRNAs (miR) are epigenetic gene regulators with tumorsuppressive or oncogenic roles in various carcinomas. This study assesses chemoresistant PDAC for its specific miR expression pattern.

**Methods:**

Gemcitabine-resistant variants of two mutant p53 human PDAC cell lines were established. Survival rates were analyzed by cytotoxicity and apoptosis assays. Expression of 1733 human miRs was investigated by microarray and validated by qRT-PCR. After in-silico analysis of specific target genes and proteins of dysregulated miRs, expression of MRP-1, Bcl-2, mutant p53, and CDK1 was quantified by Western blot.

**Results:**

Both established PDAC clones showed a significant resistance to gemcitabine (p<0.02) with low apoptosis rate (p<0.001) vs. parental cells. MiR-screening revealed significantly upregulated (miR-21, miR-99a, miR-100, miR-125b, miR-138, miR-210) and downregulated miRs (miR-31*, miR-330, miR-378) in chemoresistant PDAC (p<0.05). Bioinformatic analysis suggested involvement of these miRs in pathways controlling cell death and cycle. MRP-1 (p<0.02) and Bcl-2 (p<0.003) were significantly overexpressed in both resistant cell clones and mutant p53 (p = 0.023) in one clone.

**Conclusion:**

Consistent miR expression profiles, in part regulated by mutant TP53 gene, were identified in gemcitabine-resistant PDAC with significant MRP-1 and Bcl-2 overexpression. These results provide a basis for further elucidation of chemoresistance mechanisms and therapeutic approaches to overcome chemoresistance in PDAC.

## Introduction

Pancreatic ductal adenocarcinoma (PDAC) is the most lethal entity among human visceral cancers with increasing incidence and mortality in the United States and Europe. PDAC is currently the fourth leading cause of cancer-related death with a 5-year overall survival rate of less than 4% and is predicted to rise to second place behind lung cancer until 2020. Despite advances in clinical management and multimodal therapeutic regimens, 6-month progression-free survival remains below 15% [1]. The advances in cancer research that have led to improved prognosis in many hematological and solid cancers could not be translated into clinical benefits for PDAC patients so far.

The poor prognosis of PDAC is mainly attributed to rapid disease progression, late diagnosis at advanced unresectable stages, and poor response to the current single first-line chemotherapeutic agent gemcitabine with objectified tumor remission in only 5–11% of patients [2]. In the absence of screening options and early clinical symptoms, early diagnosis of the disease remains unattainable. Thus, chemotherapy remains a central asset in PDAC treatment and deciphering the mechanisms underlying the disease’s high level of chemoresistance is critical.

For a decade, chemotherapy with the cytidine nucleoside analogue and ribonucleotide reductase inhibitor gemcitabine has been the gold standard in the adjuvant treatment of locally advanced PDAC. Gemcitabine is a prodrug which requires cellular uptake and intracellular phosphorylation to its active diphosphate and triphosphate metabolites that inhibit DNA and RNA replication. So far, no reliable molecular targets exist to predict or influence the success of chemotherapy with gemcitabine in PDAC.

We have previously reviewed the role of (epi-)genetic markers for chemosensitivity and chemoresistance in PDAC [3]. Specifically, we discussed the role of microRNAs (miRs), a new class of small, noncoding single-stranded RNA molecules as potential key regulators in tumor oncogenesis with oncogenic or tumor suppressive properties. In this regard, only selected miRs have thus far been investigated for a role in PDAC-chemoresistance. We hypothesized that currently unidentified miRs take part in PDAC chemoresistance and present targets for novel diagnostic and therapeutic options. In this experimental study we aimed to 1) generate stable gemcitabine-resistant variants of primary, gemcitabine-susceptible human PDAC cell lines, and 2) identify chemoresistance-specific miR expression patterns.

## Materials and Methods

### Cell lines and cell culture

The certified human caucasian PDAC cell lines MIA-PaCa-2, PANC-1, BxPC-3, SU.86.86, and AsPC-1 were purchased from the American Type Culture Collection (ATCC; Rockville, MD). The cell lines carry TP53 missense mutations, all sequenced and validated by ATCC.

PANC-1 and MIA-PaCa-2 cells were cultured in phenol red free Dulbecco's Modified Eagle Medium (DMEM; Lonza, Walkersville, USA) supplemented with 10% heat inactivated fetal calf serum (FCS; Gibco, Carlsbad, USA), 2mM L-glutamine (Gibco), and 1mM sodium pyruvate (Sigma, St. Louis, USA) in a humidified incubator containing 95% air and 5% CO_2_ at 37°C. The medium for MIA-PaCa-2 cells was also supplemented by 2% heat inactivated horse serum (Gibco). BxPC-3, SU.86.86, and AsPC-1 cells were cultured under the same conditions in phenol red free RPMI-1640 medium (Gibco) containing 10% heat inactivated FCS, 2mM L-glutamine, 1mM sodium pyruvate, and 4.5g/l glucose (Sigma). All cell culture experiments were carried out without antibiotics.

### Drug sensitivity assay

Depending on the PDAC cell line, 3500–8000 cells/well were seeded into 96-well culture plates in culture medium and allowed to attach at 37°C and 5% CO2 in humidified air for 24h. To determine the intrinsic drug sensitivity, each of the 5 parental PDAC cell lines was incubated with increasing concentrations (0.0001 to 100μM) of Gemcitabine (Eli Lilly, Bad Homburg, Germany) for 72h. Subsequently, 100μl per well of methylthiazolyldiphenyl-tetrazolium bromide (MTT) solution (5mg/ml; Sigma) was added to the cells followed by incubation for 3h. Then, 100μl of dimethyl sulfoxide (DMSO; Sigma) was substituted for the supernatant, followed by incubation for 30min. Absorbance at 570nm with background subtraction at 650nm was detected using an ELISA microplate reader (Dynatech MR5000, Dynatech Laboratories Inc., VA). The MTT assay is a sensitive method of screening for in vitro drug responsiveness with a colorimetric signal proportional to the viable cell number as indicator of chemotherapy response with high reproducibility [4–7]. All results were normalized to the corresponding controls and the relative viability was calculated. Chemosensitivity assays were carried out in quadruplicate and repeated at least three times in separate experiments.

### Establishment of chemoresistant PDAC cell clones

Gemcitabine-resistant PANC-1 (PANC-1-GR) and MIA-PaCa-2 (MIA-PaCa-2-GR) cell clones were produced by exposing the parental cells to repeated pulsatile gemcitabine treatment over 3 days with constant sublethal concentrations followed by recovery-periods with agent-free medium until the cells recovered exponentially. Parental PANC-1 cells were treated with 0.4μM gemcitabine cycles for approximately 9 months. Parental MIA-PaCa-2 cells were exposed to 0.06μM gemcitabine cycles for approximately 12 months.

Morphology, time to recovery, doubling time and degree of acquired chemoresistance, using MTT cytotoxicity assay, were investigated for PANC-1 cell clones after 16–17 and 28–29 chemotherapy cycles, and after 28–29 chemotherapy cylces for MIA-PaCa-2 cell clones. Parental and chemoresistant cell clones with the same number of culture passage were compared with each other. Light microscopy and imaging (Eclipse E1000M and NIS-Elements D3.1 imaging software, Nikon, Düsseldorf, Germany) were used to evaluate morphologic changes in parental and chemoresistant PANC-1 and MIA-PaCa-2 cell clones.

### Apoptosis assay

Parental and chemoresistant PANC-1 (1.5x10^5^) and MIA-PaCa-2 (1.2x10^5^) cells were seeded onto covered chamberslides for 24h, followed by 72h exposure to half maximal inhibitory concentration (IC_50_) of gemcitabine. PDAC cell monolayers were stained with Annexin-V-FLUOS Staining Kit (Roche, Mannheim, Germany) according to the manufacturer’s instructions. Cell viability was assessed by apoptosis assays in three independent experiments and determined under a fluorescence microscope (Eclipse E800, Nikon, Düsseldorf, Germany) by counting live, apoptotic, and necrotic cells.

### MicroRNA microarray and data analysis

Total RNA was isolated from parental and chemoresistant PDAC cell lines using Trizol reagent (Qiagen, Hilden, Germany) and miRNeasy Mini Kits (Qiagen) according to the manufacturer’s instructions. RNA concentration and purity were measured using a Nanodrop spectrophotometer (Thermo Fisher Scientific Inc., Braunschweig, Germany). RNA integrity was determined using the Agilent 2100 Bioanalyzer on a RNA 6000 Nano/Pico LabChip (Agilent Technologies, Santa Clara, CA). Investigated samples had RIN values >8.0.

Affymetrix GeneChip miRNA microarrays (Affymetrix UK Ltd., High Wycombe, UK) were performed using the manufacturers´ protocols. The samples were prepared from 1μg of total-RNA in accordance with the Affymetrix FlashTag Biotin HSR RNA Labeling Kit. The targets were hybridized overnight to Affymetrix GeneChip miRNA arrays. Following hybridization, the arrays were washed and stained using the Affymetrix GeneChip Fluidics Station 450 and scanned using the Affymetrix GeneChip Scanner 3000 7G.

Microarray data quality was checked as recommended by the manufacturer and by the quality metrics in the Partek Genomics Suite software (Partek Inc., St. Louis, MO). Statistical analyses of microarray data were performed using the Partek Genomics Suite. CEL-files (containing raw expression measurements) were imported to Partek GS. The robust multi-array average algorithm was used for normalization. The array data were quantile-normalized and log-2 transformed. For each probe, a one-way analysis of variance was performed. Fisher's least significant difference was tested to statistically compare the difference between the means of the *groups´* expression measurements. A *p*-value <0.05 was used as a threshold for significance. Microarray raw data are available through Gene Expression Omnibus accession number GSE74574 (www.ncbi.nlm.nih.gov/geo).

### Quantitative real time PCR

Quantitative Real-Time (qRT) PCR was performed using the miScript PCR system (Qiagen). Total RNA samples (1μg) of PDAC cell lines were reverse transcribed to cDNA using miScript Reverse Transcription Kits (Qiagen). For each sample, 5μL of the generated cDNA was mixed with 10μL 2x QuantiTect SYBR, 2μL 10x miScript Universal Primer, 2μL gene specific 10x miScript Primer Assay and 1μL nuclease free water. All samples were analyzed in triplicate reactions using Applied Biosystems 7900HT Real-Time PCR System (Applied Biosystems, Darmstadt, Germany) with miScript SYBR Green PCR Kit (Qiagen). The cycling program involved preliminary denaturation at 95°C for 10min, followed by 40 cycles of denaturation at 95°C for 15s, annealing at 60°C for 60 s and elongation at 60°C for 60s. Quantitative miR analysis was performed using SDS-Software v2.3 (Applied Biosystems). Expression of each miR was analyzed quantitatively relative to the housekeeping genes RNU6-2, SNORD68, and SNORD96A by the 2^–ΔΔCT^ method.

### Target identification and Western blot analyses

Potential gene targets or regulators of the detected miRs were identified by literature review and in silico, using Ingenuity Pathway Analysis (IPA) and DIANA-mirPath. Based on this preselection we focused on protein targets that are commonly known to be relevant for cellular resistance to chemotherapy. Due to its role as `classic`drug resistance marker, MRP-1 expression was investigated after generation of chemoresistant PDAC cell clones. Monoclonal antibodies against human MRP-1 (QCRL1; abcam, 1:100; 1.5μg/ml), p53 (1C12; Cell Signaling Technology, 1:1000), CDK1 (A17; abcam, 1:500; 2μg/ml), Bcl-2 (AW604; Millipore, 1:1000; 1μg/ml), and actin (Sigma, 1:2000; 0.35μg/ml) were used as primary antibodies.

The employed p53 antibody detects wild-type p53 as well as mutant-p53. The p53 status of both human PDAC cell lines was sequenced and validated by ATCC. Accordingly, MIA-PaCa-2 and Panc-1 are homozygous mutant p53 cell lines that carry hotspot mutations in the codons 248 and 273, respectively. Therefore, the p53 analysis allows the detection of p53 R273H expression in PANC-1 and p53 R248W in MIA-PaCa-2 cell lines [8].

Total cell lysates were obtained by incubation in lysis buffer for 1h at 4°C. After centrifugation at 14000 g, supernatant proteins were electrophoretically separated on 7.5% SDS-PAGE, loaded with equal amounts of protein. Following electrophoresis, the proteins were transferred to a nitrocellulose membrane (Amersham Life Sciences). The primary antibodies were added in the appropriate dilution and incubated overnight at 4°C. The nitrocellulose membranes were washed in PBS (pH 7.4) containing 0.05% Tween20 and incubated with the secondary antibody anti-mouse IgG-HRP (Sigma, 1:13000; 0.08μg/ml) for 1h at room temperature, followed by a repeated Tris-buffered saline (without Tween20) wash. Antibody binding signal was detected using enhanced chemiluminescence (Millipore, Schwalbach, Germany), and protein quantification and band intensities were analyzed using ImageJ densitometry software (version 1.46r, NIH, USA). Band intensity was determined by area under the curve and calibration with standards on the same blot.

### Statistical analysis

Statistical analysis was performed with the Statistical Package for the Social Sciences software program, version 17.0 (SPSS inc., Munich, Germany) for Windows®. Data were expressed as means of normalized expression. Statistical significance was determined by using the two-tailed Student’s t-test to compare two data sets. *P*<0.05 was considered to be statistically significant.

## Results

### Intrinsic chemoresistance in human parental PDAC cell lines

**Fig 1:**
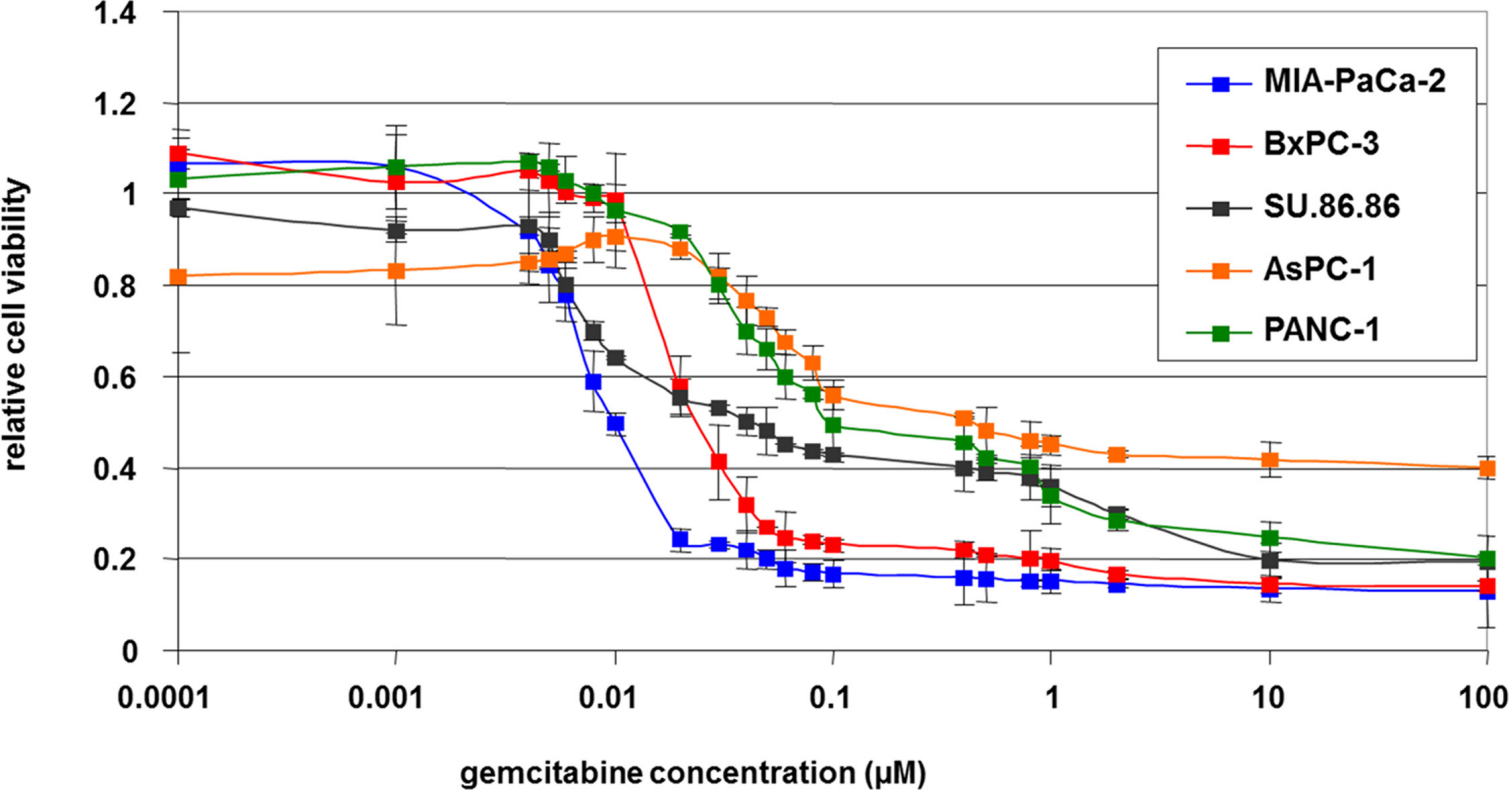
Intrinsic drug sensitivity of parental PDAC cell lines. Relative cell viability of five human PDAC cell lines following 72 h exposure to ascending concentrations of gemcitabine by MTT cytotoxicity assay.

The therapeutic index was determined by MTT cytotoxicity assay using the dose-response curves of five human PDAC cell lines with IC_50_ and sublethal doses after 72h of gemcitabine chemotherapy (Fig 1). IC_50_ was recorded within a range of 0.01 to 0.8μM. Although the gemcitabine treatment caused a concentration dependent inhibition of growth in all 5 PDAC cell lines, MIA-PaCa-2 tended to be the cell line with the highest intrinsic chemosensitivity, whereas PANC-1 and AsPC-1 were the cell lines with the most distinct intrinsic chemoresistance. For further generation of acquired drug resistant cell clones, we selected MIA-PaCa-2 and PANC-1, two well-known primary PDAC cell lines of different intrinsic chemoresistance with an IC_50_ of 0.01μM *vs*. 0.1μM.

### Generation of extrinsic chemoresistance in human PDAC cell lines

**Fig 2:**
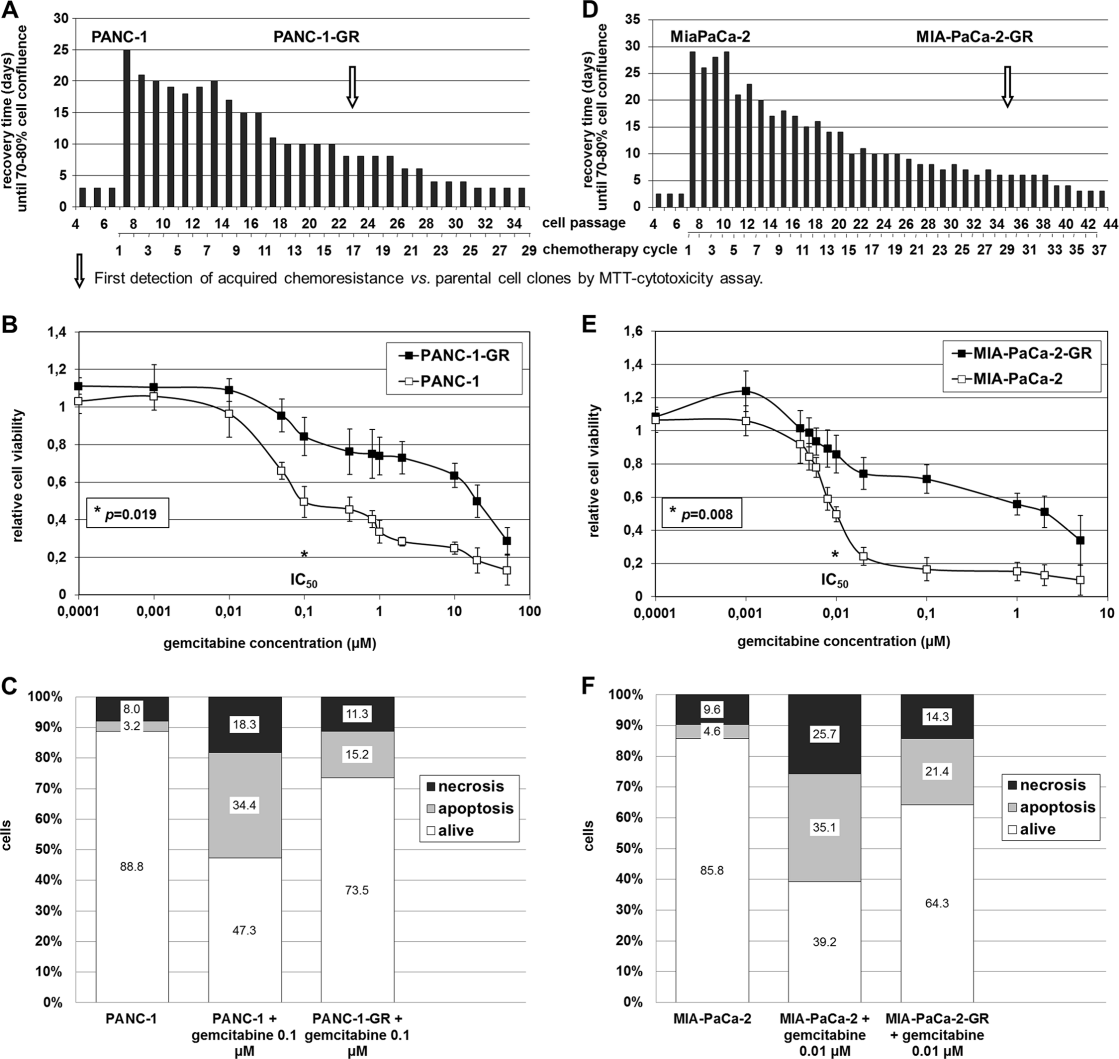
Establishment of chemoresistant PDAC cell clones. Generation of chemoresistant PDAC cell clones by repeated pulsatile treatment over 3 days with constant sublethal concentrations of 0.4μM (A) or 0.06μM (D) gemcitabine followed by recovery-periods and quantification of cell viability by MTT assay as well as apoptosis assay in parental vs. chemoresistant PANC-1 (A, B, C) and MIA-PaCa-2 (D, E, F) cell clones.

**Fig 3:**
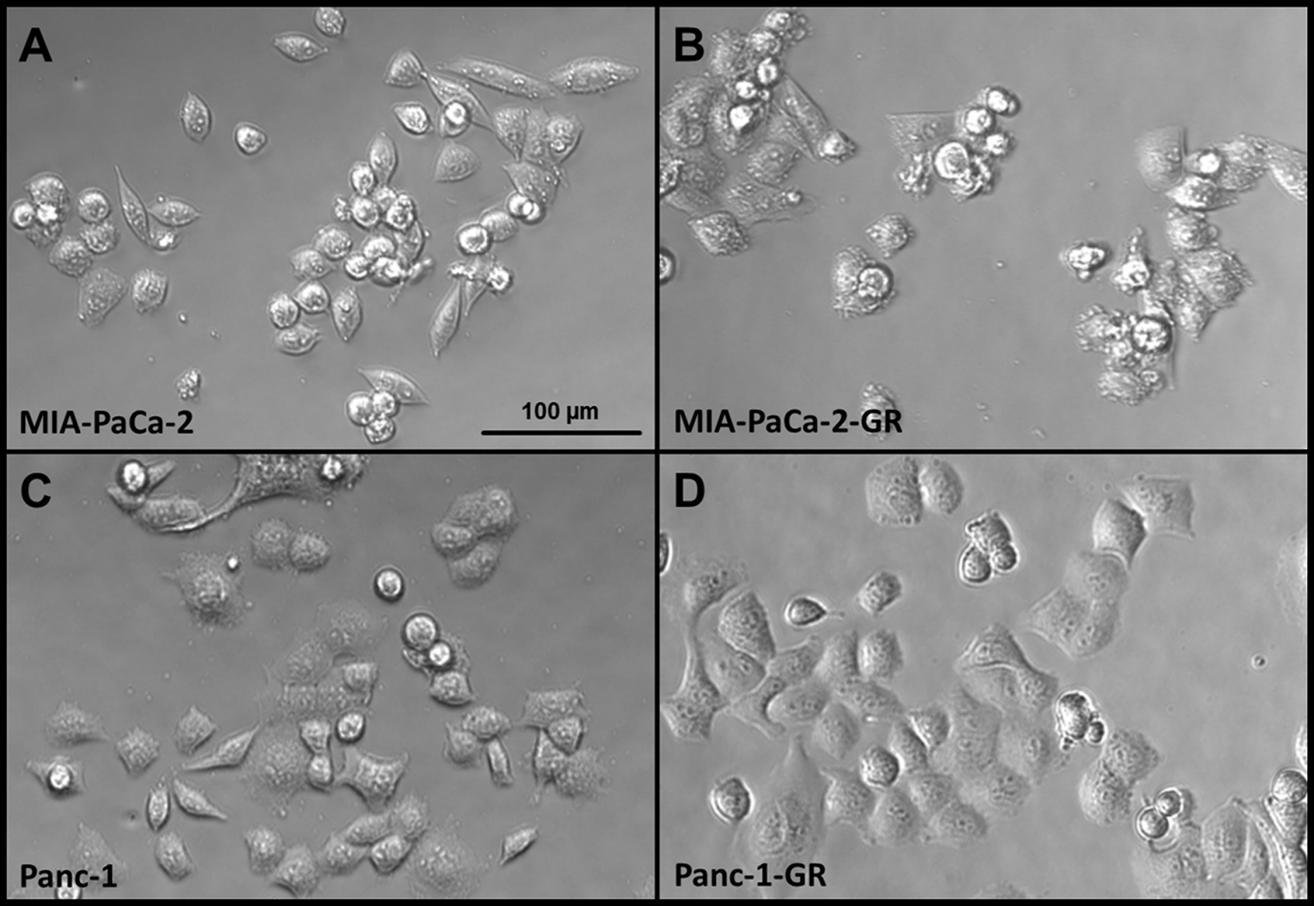
Morphologic changes in parental and chemoresistant PANC-1 and MIA-PaCa-2. Representative attached epithelial cells with spindle-shaped cells in untreated MIA-PaCa-2 (A) and PANC-1 cells (B) compared to more plump rounded morphology and enhanced formation of pseudopodia in their chemoresistant cell clones (C, D).

Generation of PDAC cell clones with acquired chemoresistance was achieved after 17 chemotherapy cycles in PANC-1 and after 29 cycles in MIA-PaCa-2 cells. Stably resistant PDAC cell clones were established following repeated pulsatile treatment over 3 days with constant sublethal concentrations of gemcitabine over a 9 to 12 month period (Fig 2A and 2D). MTT cytotoxicity assay revealed a significantly improved cell viability of gemcitabine treated PANC-1-GR (*p* = 0.019) and MIA-PaCa-2-GR cells (*p* = 0.008). Compared with their parental cells, the IC_50_ of the generated chemoresistant cell clones was about 150 to 200 times higher (Fig 2B and 2E). Concordantly, cell death analysis by Annexin-V / Propidium iodide fluorescence staining showed decreased apoptosis and necrosis of IC_50_ gemcitabine treated PANC-1-GR and MIA-PaCa-2-GR cell clones compared to their parental cells of 26.5 ±3.4% *vs*. 52.7 ±2.9% (*p*<0.001), respectively 35.7 ±3.5% *vs*. 60.8 ±7.4% (*p* = 0.01) (Fig 2C and 2F). Prolonged treatment of PANC-1 and MIA-PaCa-2 cells with gemcitabine resulted in morphologic changes with more plump, rounded cell morphology in MIA-PaCa-2-GR and PANC-1-GR cell clones compared with untreated PDAC cells with spindle-shaped character and enhanced formation of pseudopodia (Fig 3).

### MicroRNA-expression profiling of chemoresistant human PDAC cell lines

**Fig 4:**
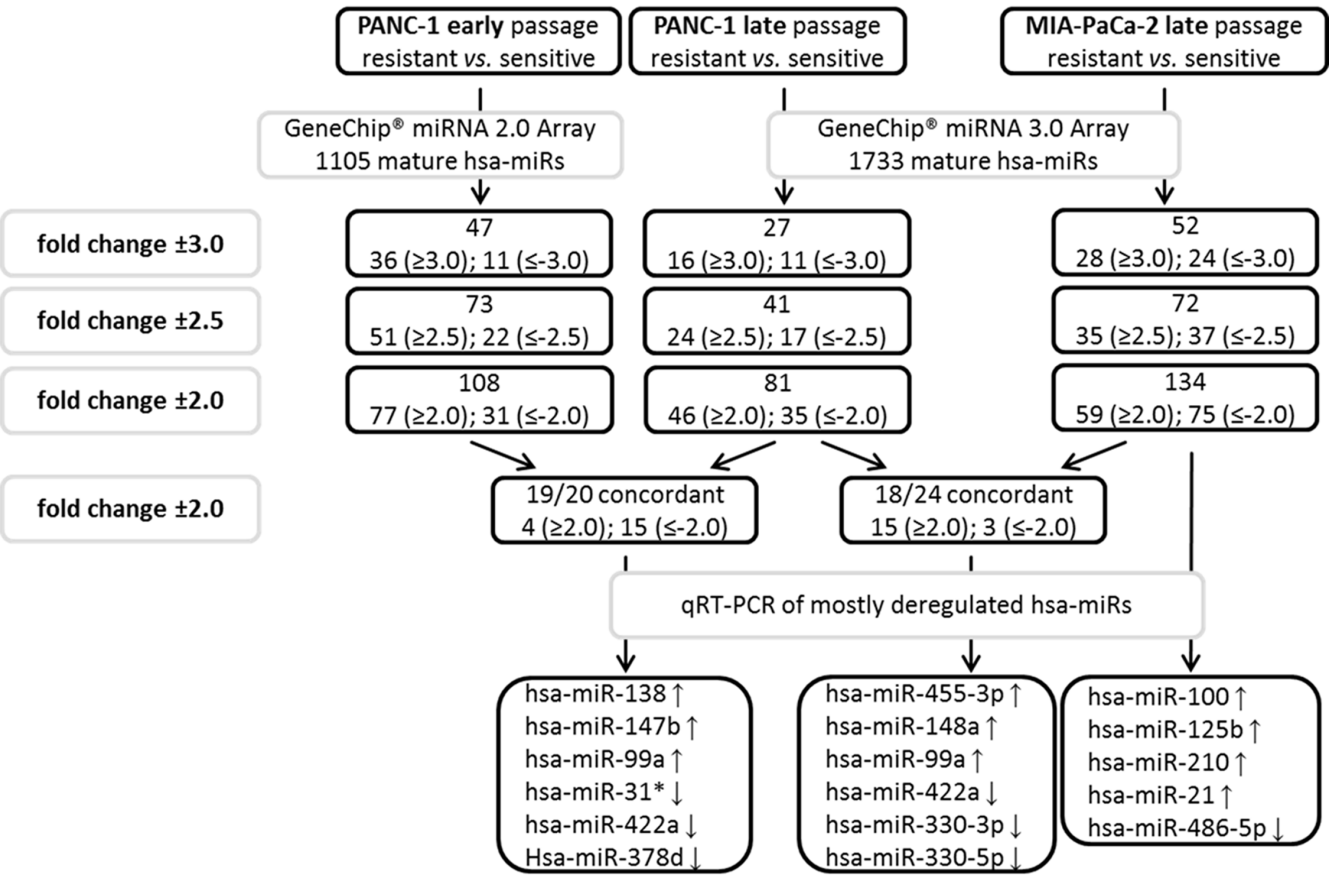
MicroRNA profiling. Procedure and results of miR expression profiling by GeneChip microarray and qRT-PCR validation in parental and gemcitabine resistant PANC-1 and MIA-PaCa-2 cell clones.

**Fig 5:**
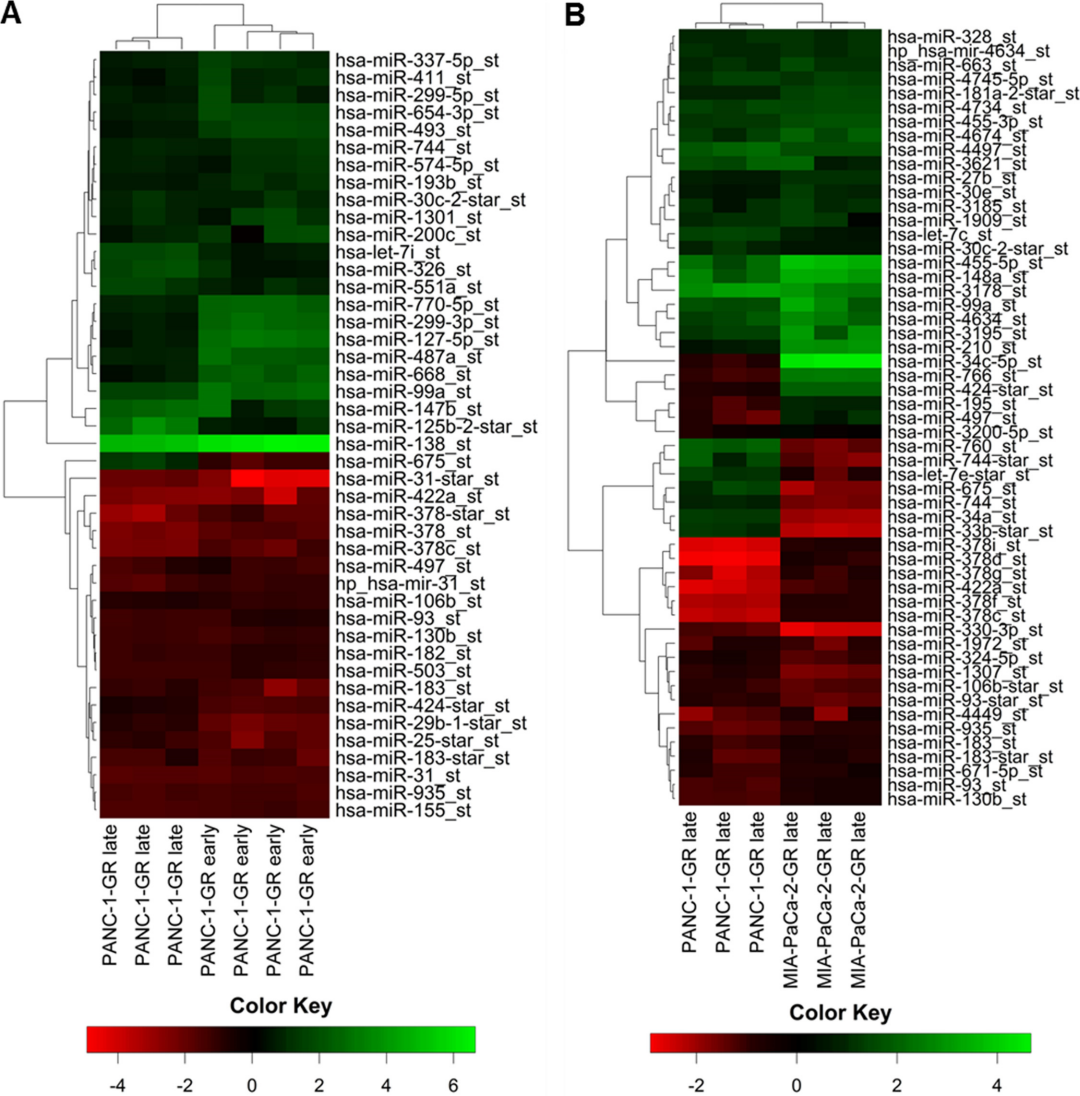
MicroRNA heat mapping. Hierarchical cluster heat maps of significantly dysregulated miRs in PANC-1-GR late and early cell passage (A) as well as in PANC-1-GR and MIA-PaCa-2-GR late cell passage (B). Each row shows the relative expression level for a single miR and each column shows the expression level for a single sample; fold change greater than ±2 (p<0.05). The red or green color indicates low or high expression, respectively.

**Fig 6:**
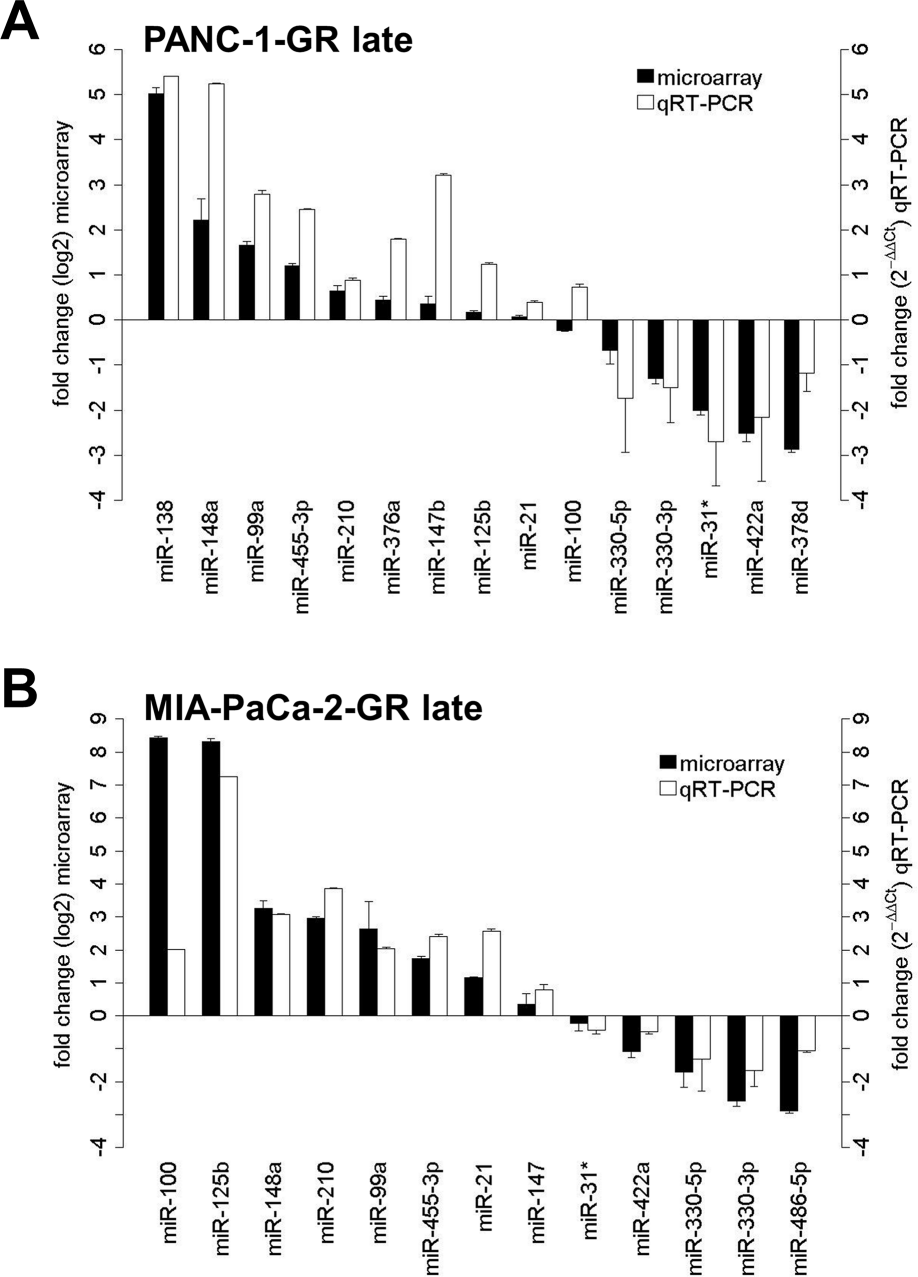
MicroRNA microarray validation. MiR microarray validation with qRT-PCR for significantly dysregulated miRs in PANC-1-GR (A) and MIA-PaCa-2-GR cell clones (B). Fold change from miR microarray is demostrated by log2 values (left y-axis, dark grey bars). Fold change from qRT-PCR was determined using the 2^-ΔΔCt^ method (right y-axis, light grey bars). Error bars represent the standard deviation of mean.

GeneChip miR microarray was finally performed in parental and chemoresistant PANC-1 cells after 17 and after 29 chemotherapy cycles. Comparing the profiles of significantly dysregulated miRs in early and late cell passage of PANC-1 cells with acquired chemoresistance, a miR panel of high congruence with 19 concordantly expressed miRs (fold change ±2) was identified (Fig 4). In hierarchical clustering using Manhattan distance and average linkage, PANC-1-GR cell clones with early and late acquired chemoresistance clustered together on the dendrogram with an accordance of significantly dysregulated and concordantly expressed miRs of more than 95%. No significant change of miR profile in early and late chemoresistance of PANC-1-GR cell clones was detected (Fig 5A). RT-PCR validation of mostly dysregulated miRs confirmed that miR-138, miR-147b, miR-148a, miR-99a, miR-455-3p and miR-125b were significantly upregulated and miR-31-star, miR-422a, miR-330-3p, mir-330-5p and miR-378d were downregulated in PANC-1-GR cell clones vs. their parental cell line (Fig 6A).

Similarly, miR expression profiling in MIA-PaCa-2-GR cell clones was performed but limited to late passage cells due to the nearly identical results between early and late cycle cells in PANC-1-GR (Fig 5B). In MIA-PaCa-2-GR cell clones miR-125b, miR-210, miR-21, miR-100, miR-148a, miR-99a and miR-455-3p were significantly upregulated, whereas miR-330-3p, miR-330-5p, miR-486-5p, miR-422a and miR-31-star were significantly downregulated (Fig 6B). Further significantly dysregulated, but not yet functionally characterized miRs in both PANC-1-GR as well as MIA-PaCa-2-GR cell clones were not validated by qRT-PCR (miR-935, miR-3178, miR-3195, miR-4497, miR-4634, miR-4734, and miR-4745-5p). Overall, a panel of 18 significantly and concordantly dysregulated miRs (fold change ±2; *p*<0.05) in both chemoresistant PDAC cell clones was identified.

### Target analysis and protein expression of MRP-1, p53, CDK1, and Bcl-2

**Fig 7:**
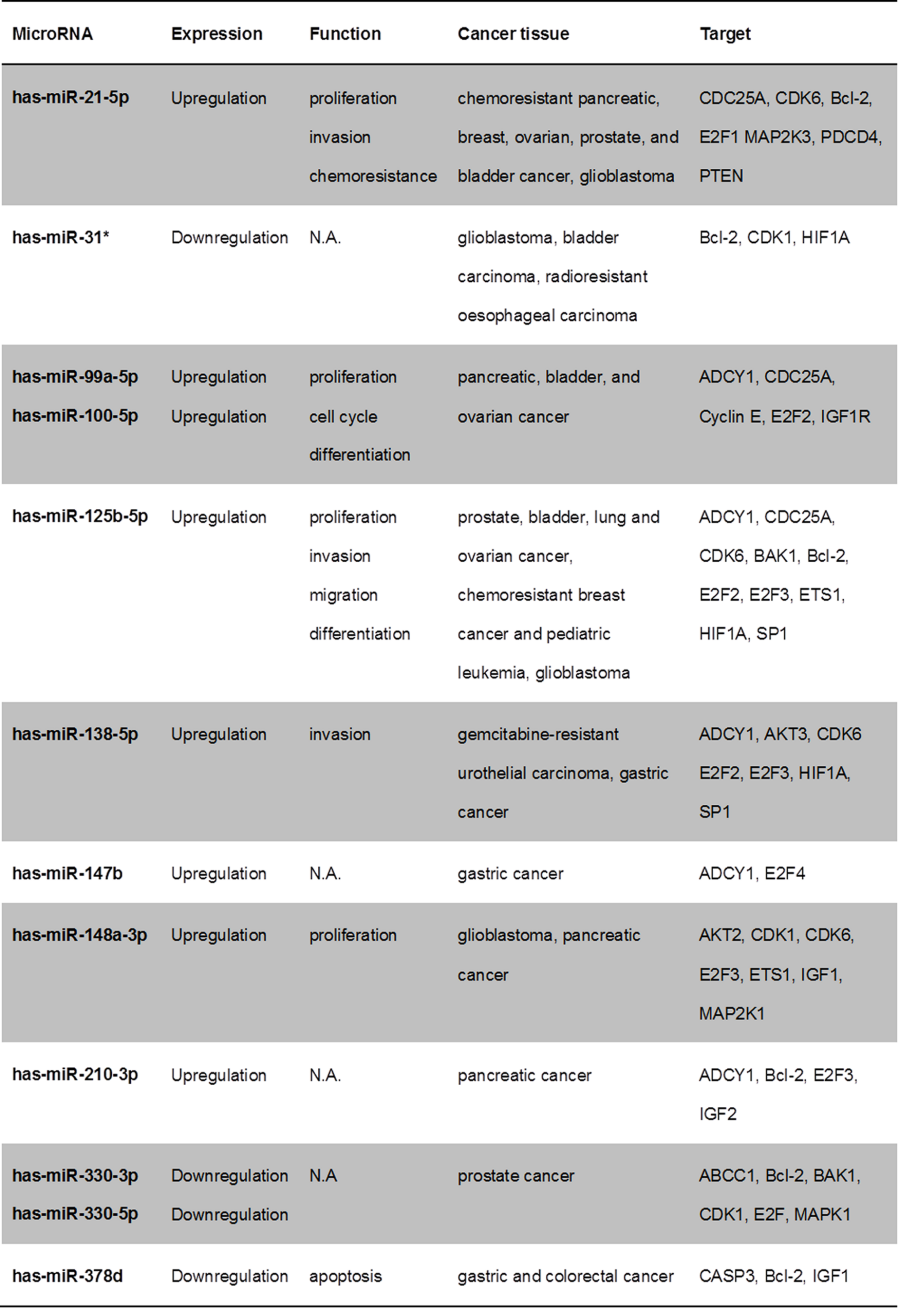
In-silico target analysis. Overview of selected and highly predicted gene targets of dysregulated miRs in chemoresistant PDAC cell clones. (N.A., not available)

In-silico target analysis of significantly dysregulated miRs in PANC-1-GR and MIA-PaCa-2-GR and of mt-p53 identified regulators of cell cycle, proliferation, and apoptosis (Fig 7). MiR-21-5p, miR-100-5p, miR-125b-5p, miR-210-3p, miR-330-3p, miR-378a-3p, and miR-486-5p are predicted by IPA to be regulated by TP53 gene. Multidrug resistance proteins like MRP-1 (ABCC1) are highly predicted to be regulated by mt-p53, but also by miR-330-5p. Apoptosis and cell cycle regulators like Bcl-2 and CDK-1 are predicted to be regulated by most of the dysregulated miRs in chemoresistant cell clones and by mt-p53.

**Fig 8:**
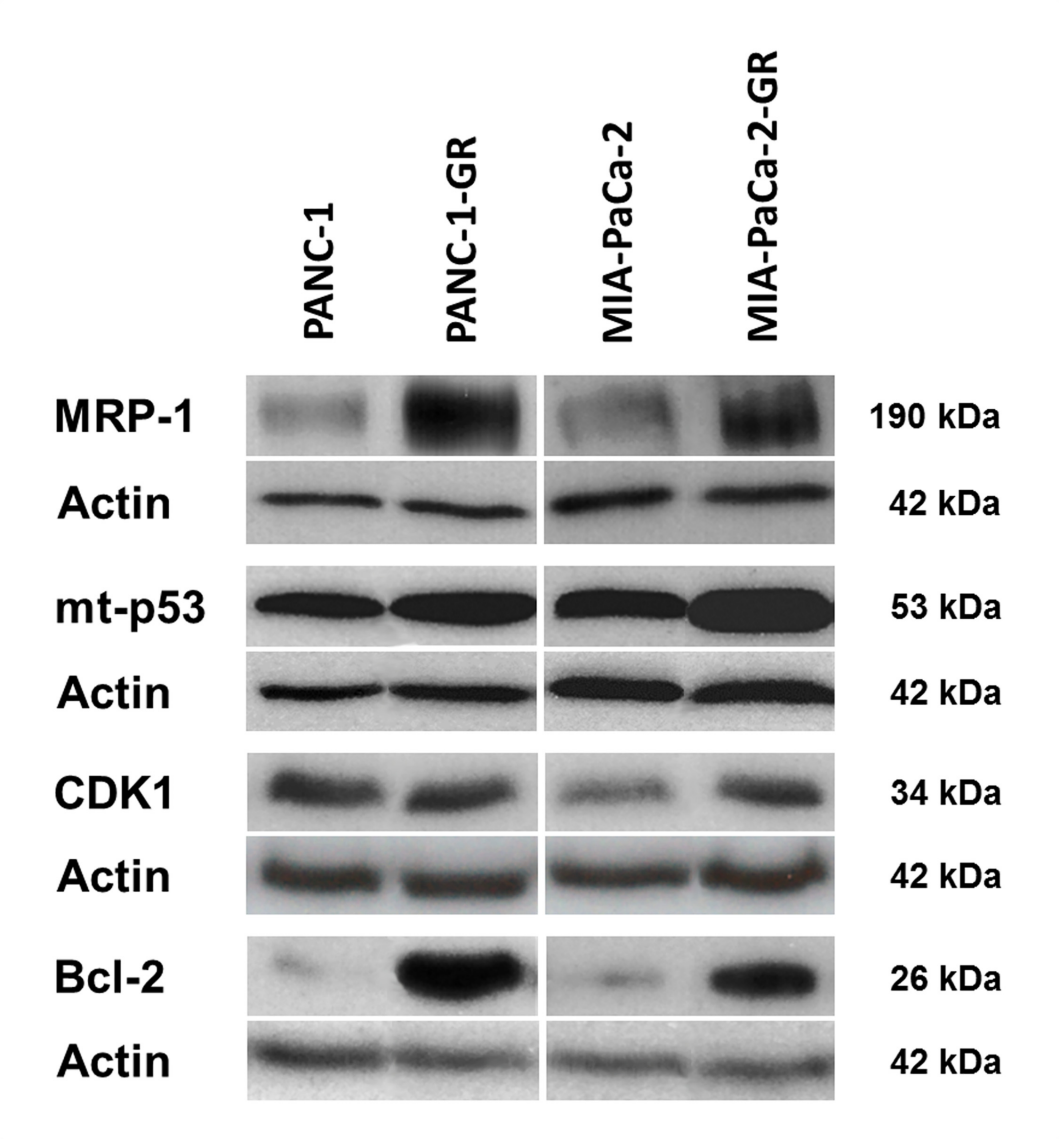
Protein target expression. Protein expression of MRP-1, mutant p53 (mt p53 R273H and mt p53 R248W), CDK1, Bcl-2, and actin (loading control) in parental (PANC-1, MIA-PaCa-2) and gemcitabine resistant PDAC cell clones (PANC-1-GR, MIA-PaCa-2-GR) by Western blot.

**Fig 9:**
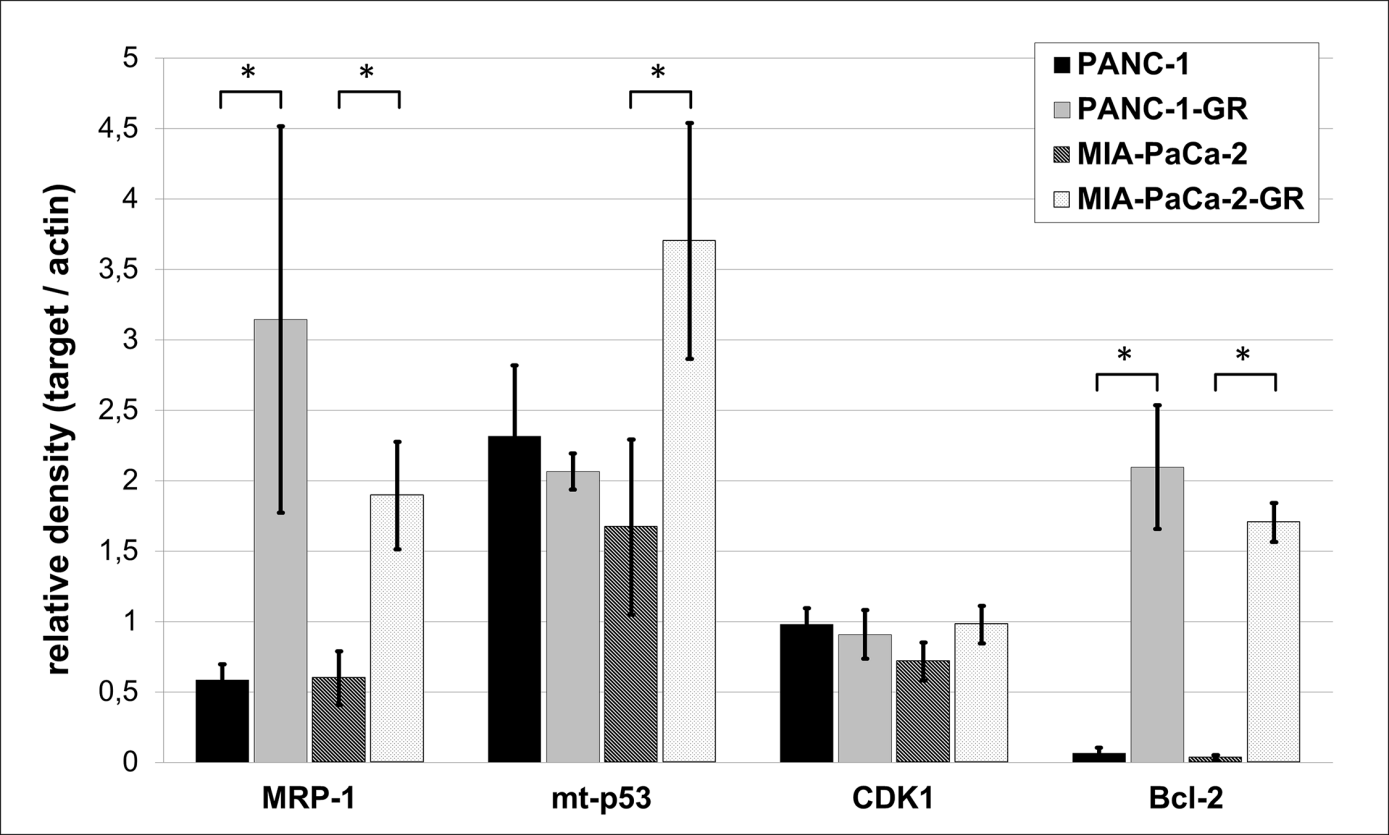
Protein densitometry. Adjusted relative density of MRP-1, mutant p53 (mt p53 R273H and mt p53 R248W), CDK1, Bcl-2, and their loading controls measured by ImageJ densitometry software. Asterisks indicates to a significant difference of p<0.05, respectively.

The expression of three target proteins that were selected from the in-silico analysis (p53, CDK1, Bcl-2) and the `classic`drug resistance marker MRP-1 were evaluated in parental and chemoresistant cell clones by Western blot (Figs 8 and 9). MRP-1 was significantly overexpressed in both PANC-1-GR and MIA-PaCa-2-GR cell clones (*p* = 0.017; *p* = 0.006), supporting its property as a marker for drug resistance. The miR-target and apoptosis suppressor Bcl-2 was significantly overexpressed in both PANC-1-GR and MIA-PaCa-2-GR cells (*p* = 0.002; *p*< 0.0001). High expression of mt-p53 was shown in both chemoresistant cell clones, but significance was only achieved in MIA-PaCa-2-GR (*p* = 0.023). Expression of the miR- and mt-p53- target and cell cycle regulator CDK1 was increased in MIA-PaCa-2-GR cells but did not reach statistical significance (*p* = 0.063).

## Discussion

Chemoresistance to PDAC treatment represents both a clinical and scientific challenge. After initial response, gemcitabine treated PDAC patients finally exhibit disease progression owing to acquired chemoresistance. Therefore, several combinations of gemcitabine and gemcitabine free regimens have been evaluated but very few showed additional benefits with a slight increase in overall survival [9–14]. Understanding primary and secondary chemoresistance is therefore crucial for further clinical developments.

Human PDAC cell lines possess high intrinsic chemoresistance. The method of establishing novel chemotherapy-resistant PDAC cell clones by pulsatile treatment with short periods of exposure and high sublethal concentrations of gemcitabine seems to be an experimental setting which is comparable to the clinical relevant treatment regimens. It applied selective pressure for PANC-1 and MIA-PaCa-2 cell clones with intrinsic mutations, already conferring a degree of chemoresistance. We used the MTT cytotoxicity assay as an established in vitro method to test malignant cell resistance to clinically applied cytostatics [4–7]. It remains unclear whether these in vitro mechanisms of chemoresistance can be directly translated to the clinical setting. In the future, microdissected tumor cells of the same PDAC patients before and after gemcitabine treatment may be recovered and analyzed to identify clinically relevant chemotherapy response markers.

While the crucial role of miRs as key regulators of carcinogenesis has been recognized, little is known about their significance and clinical relevance in regard to chemoresistance.

In the present study, we have for the first time investigated the entire currently known human mature miR spectrum in two of the most studied human PDAC cell lines and their corresponding chemoresistant clones generated by pulsatile gemcitabine treatment with constant treatment dose. Concordant miR profiles were aberrantly expressed in both chemoresistant PDAC cell lines, discriminating them from their parental cell lines. Some of these miRs, such as miR-21, miR-99a, miR-100, and miR-210 are already known as potential oncogenes (oncomiRs) in PDAC. Specifically, miR-21 has been shown to be associated with poorer survival and to induce chemoresistance to gemcitabine in PDAC patients [15]. In smaller clinical studies, miR-210 has been reported to be overexpressed in PDAC patients, correlating with a worse outcome [16]. MiR-99a and miR-100, two members of the miR-99 family, were found by Bloomston *et al*. to be overexpressed in PDAC tissue compared with normal pancreatic tissue and chronic pancreatitis [17]. Recently, *in vitro* miR analysis was performed by Bera *et al*. in BxPC-3 PDAC cells with acquired chemoresistance by gemcitabine treatment with increasing concentrations over a six-week period [18]. In accordance with our results, miR-21, miR-100, and miR-125b were upregulated, whereas miR-455-3p and miR-378 were downregulated in chemoresistant BxPC-3. In addition, miR-330-5p could be detected by Tréhoux et al. as a tumor suppressor in PDAC in vitro and in vivo, sensitizing pancreatic cancer cells to gemcitabine [19]. Other dysregulated miRs identified in the present study have not been described in PDAC but in other carcinoma entities, so far (Fig 7) [20–40].

It appears that the selection of human PDAC cell lines and the manner of drug exposure to generate PDAC cell clones with acquired chemoresistance greatly influence molecular mechanisms of chemoresistance development with impact on the miR profile. In contrast to matching results with Bera *et al*., miR screening in PDAC cell lines with induced gemcitabine chemoresistance by further study groups revealed different miR profiles that did not correspond to our results. For example, Shen et al. established a chemoresistant cell clone of a wild-type p53 PDAC cell line derived from a spleen metastasis generated after 7 months of continuous gemcitabine induction with increasing concentrations. Iwagami et al. generated chemoresistant cell clones of two mutant p53 primary PDAC cell lines by exposure to gradually increasing concentrations of gemcitabine for 2 months.[18, 41, 42] Based on the analysis of different in vitro models of chemoresistance by Watson et al. the used method of pulsatile gemcitabine treatment represents a much better approximation to the clinical therapy practice with cyclic therapy protocol than the commonly used experimental continuous drug exposure with increasing drug concentrations [43].

Approximately 75% of PDAC patients have TP53 alterations [44]. Therefore, we used two human PDAC cell lines with two of the most frequent hotspot TP53 mutations in the codons 248 (MIA-PaCa-2) and 273 (PANC-1), that do not only lose the wild-type p53 tumor suppressive function, but also gain new oncogenic properties. Through this gain of function (GOF), mutant p53 is believed to actively contribute to cancer development, progression and metastasis in PDAC [44, 45]. Many GOF effects of mutant p53 rely on its ability to bind and inactivate the p53 family members p63 and p73, leading to reduced apoptosis in tumor cells and increased chemoresistance [46]. Overexpression of mutant p53 was demonstrated together with induction of various cell cycle and proliferation associated genes in response to DNA damage [47–49]. Recently, Fiorini *et al*. showed that gemcitabine treatment stabilized mutant p53 protein in the nuclei of PANC-1 cells and induced chemoresistance, concurrent with the mutant p53-dependent expression of CDK1 and cyclin B1, resulting in hyperproliferation [50]. In accordance with these results, we were able to show an overexpression of mutant p53 and its target protein CDK1 in chemoresistant MIA-PaCa-2 as well as an overexpression of anti-apoptotic Bcl-2 in both chemoresistant cell clones. Inversely, adenoviral vector transfection of the wild-type p53 tumor suppressor gene by Bouvet *et al*. in mutant p53 MIA-PaCa-2 and PANC-1 induced apoptosis and inhibited tumor cell growth [51]. Moreover, knockdown of endogenous mutant p53 rendered cancer cells more sensitive to DNA-damaging chemotherapeutic agents and reduced tumor malignancy [52]. In 1998 Wada *et al*. could induce apoptosis with Bcl-2 downregulation in mutant p53 PDAC cell lines by using a CDK- inhibitor.[53] Consistent with our results, loss of proapoptotic wild-type p53 activity and overexpression of oncogenic mutant p53 lead to transcriptional activation of multidrug resistance proteins, like the ATP-binding cassette transporter and drug eliminator MRP-1 [49, 54]. So far, multidrug resistance proteins were not described as targets of the identified chemoresistance-related miRs of this study. The role of microRNA-330-5p, significantly downregulated in both chemoresistant cell clones and highly predicted to target MRP-1 (ABCC1) needs to be confirmed by functional analysis.

**Fig 10:**
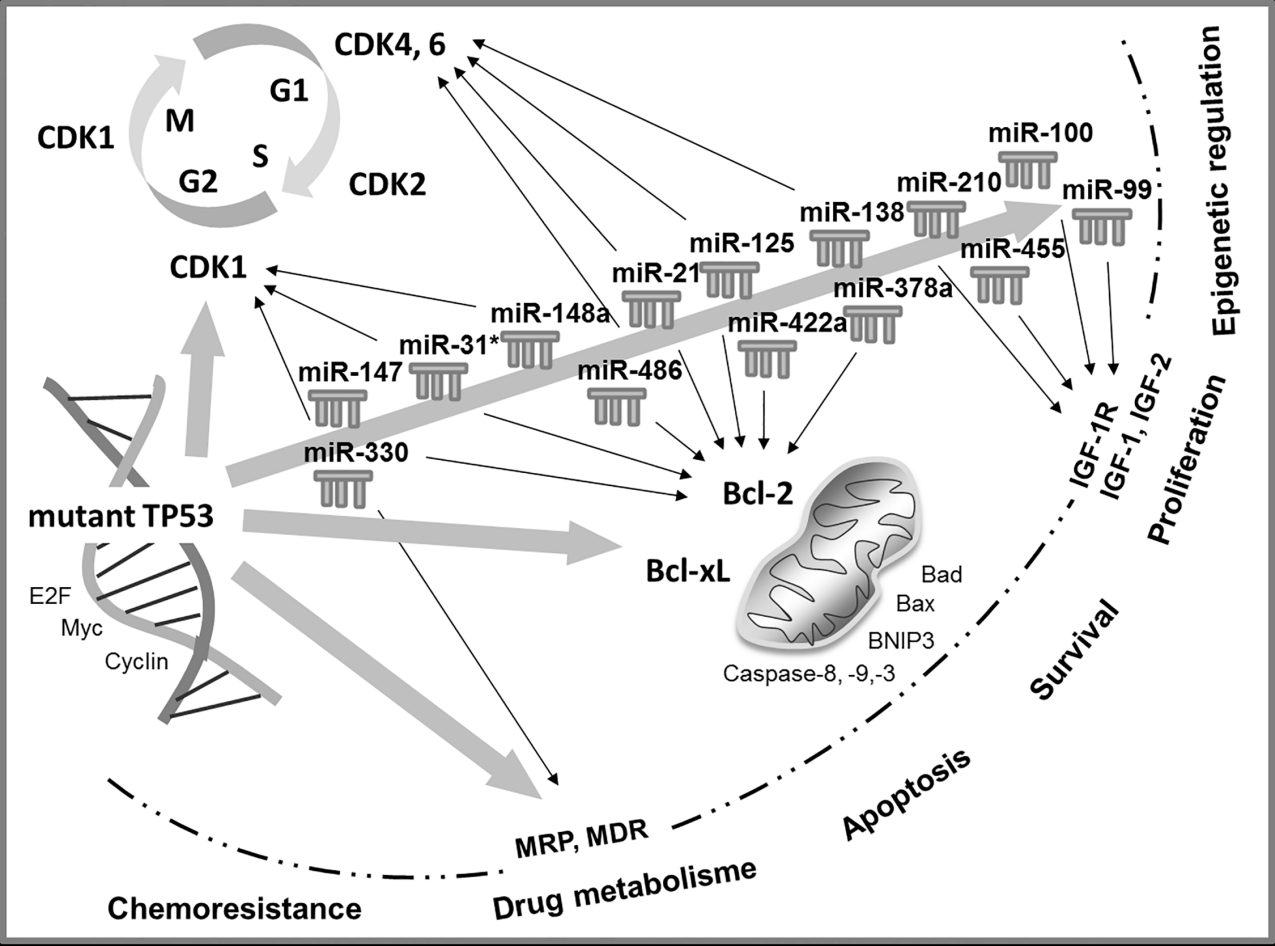
Interaction of mutant TP53 and microRNA signaling pathways in chemoresistant pancreatic cancer. Target analysis was performed by Ingenuity Pathway Analysis and DIANA-mirPath.

Moreover, recent studies reported that mutant p53 proteins can modulate miR expression [46, 55]. Interestingly, most of our chemoresistance-specific miRs (miR-21-5p, miR-100-5p, miR-125b-5p, miR-210-3p, miR-330-3p, miR-378a-3p, miR-486-5p) are predicted by IPA to be regulated by TP53 gene (Fig 10). Moreover, the predicted targets of these dysregulated miRs are regulators of cell cycle and proliferation or apoptosis (Fig 7). The cell division cycle 25 homolog A (CDC25A), required for progression from G1 to S phase of the cell cycle by activating cyclin-dependent kinases (CDK), is highly predicted to be targeted by miR-21, miR-99a, miR-100, and miR-125b [56]. In addition CDC25A is a target of the E2F family of transcription factors, targeted by most of the detected miRs in chemoresistant PDAC cell clones [57]. E2F1, E2F2 and E2F3a are activators of the cell cycle targeting cyclins, CDKs, DNA repair and replication proteins. Furthermore, cyclins, CDK4, CDK6, ETS1, and the G1 cell cycle phase specific transcription factor SP1 are activated by the mitogen-activated protein kinase (MAPK) signaling pathway, targeted by several dysregulated miRs [58]. The tumor suppressor genes programmed cell death 4 (PDCD4) and phosphatase and tensin homolog (PTEN), also involved in the regulation of cell cycle, are known to be targeted and repressed by miR-21 [15, 59]. Insulin-like growth factor (IGF) and its receptors are further highly predicted targets, overexpressed in most cancer tissues and function as anti-apoptotic regulators. In PDAC IGF1 mediates PTEN suppression and, together with IGF1R, enhances cell invasion and proliferation as well as chemoresistance via activation of the phosphatidylinositol 3-kinase (PI3K)/Akt signaling pathway [60–62]. Akt promotes growth factor-mediated cell survival by inhibiting pro‑apoptotic proteins such as caspase 9 and BAD. Most of identified chemoresistant-specific miRs target transcription of proapoptotic (BAX, BAK, BAD) and anti-apoptotic (Bcl-2) members of the Bcl-2 family. Dong *et al*. could demonstrate that direct induction of Bcl-2 by miR-21 was associated with proliferation and chemoresistance of MIA-PaCa-2 cells [63]. Our results confirm the role of Bcl-2 as apoptosis-suppressor and oncoprotein, significantly overexpressed in chemoresistant MIA-PaCa-2 and PANC-1 cell clones.

We suggest that under acquired chemoresistance, accumulation of mutant p53 induces expression of miR-21-5p, miR-31*, miR-125b-5p, miR-210-3p, miR-330-3p, miR-378a-3p, miR-422a and miR-486-5p which in turn enhances proliferation by upregulating Bcl-2 expression in PDAC cells.

Taken together, we have identified a panel of miRs associated with induced chemoresistance in PDAC. Target analysis of the dysregulated miRs showed that mutant p53 and the putative miR-targets Bcl-2 and MRP-1 are likely to play a central role in PDAC chemoresistance. However, additional functional validation of deregulated miRs and their protein targets in context of gemcitabine resistance is required to confirm our findings. Furthermore, clinical studies are essential to evaluate whether the identified miR signature of acquired gemcitabine resistance can be used to develop strategies for targeted therapies in chemorefractive PDAC patients.
